# Insight into synthesis and characterisation of Ga_0.9_Fe_2.1_O_4_ superparamagnetic NPs for biomedical applications

**DOI:** 10.1038/s41598-023-45285-y

**Published:** 2023-10-24

**Authors:** Amalia Mesaros, Alba Garzón, Mircea Nasui, Rares Bortnic, Bogdan Vasile, Otilia Vasile, Florin Iordache, Cristian Leostean, Lelia Ciontea, Josep Ros, Ovidiu Pana

**Affiliations:** 1 Physics and Chemistry Department, Technical University of Cluj-Napoca, 28 Memorandumului Street, Cluj-Napoca, Romania; 2 Institut Català de Nanocència i Nanotecnologia (ICN2), Av. Serragalliners S/N, 08193 Bellaterra, Spain; 3 Research Center for Advanced Materials, Products and Processes, National University for Science and Technology Politehnica Bucharest, Splaiul Independentei 313, S6, Bucharest, Romania; 4 National University for Science and Technology Politehnica Bucharest, National Research Center for Micro and Nanomaterials, Splaiul Independentei 313, S6, Bucharest, Romania; 5 Faculty of Veterinary Medicine, University of Agronomical Sciences and Veterinary Medicine, 105 Blvd. Splaiul Independentei, 050097 Bucharest, Romania; 6 National Institute for Research and Development of Isotopic and Molecular Technologies, 67-103 Donat Street, 400293 Cluj-Napoca, Romania; 7 Departament de Química Inorgànica, Universitat Autònoma de Barcelona, 08193 Bellaterra, Spain

**Keywords:** Materials science, Nanoscience and technology

## Abstract

A Ga^3+^-substituted spinel magnetite nanoparticles (NPs) with the formula Ga_0.9_Fe_2.1_O_4_ were synthesized using both the one-pot solvothermal decomposition method (TD) and the microwave-assisted heating method (MW). Stable colloidal solutions were obtained by using triethylene glycol, which served as a NPs stabilizer and as a reaction medium in both methods. A narrow size distribution of NPs, below 10 nm, was achieved through selected nucleation and growth. The composition, structure, morphology, and magnetic properties of the NPs were investigated using FTIR spectroscopy, thermal analysis (TA), X-ray diffraction (XRD), transmission electron microscopy (TEM), X-ray photoelectron spectroscopy (XPS), and magnetic measurements. NPs with the expected spinel structure were obtained in the case of the TD method, while the MW method produced, additionally, an important amount of gallium suboxide. The NPs, especially those prepared by TD, have superparamagnetic behavior with 2.02 μB/f.u. at 300 K and 3.06 μB/f.u. at 4.2 K. For the MW sample these values are 0.5 μB/f.u. and 0.6 μB/f.u. at 300 K and 4.2 K, respectively. The MW prepared sample contains a secondary phase and very small NPs which affects both the dimensional distribution and the magnetic behavior of NPs. The NPs were tested in vitro on amniotic mesenchymal stem cells. It was shown that the cellular metabolism is active in the presence of Ga_0.9_Fe_2.1_O_4_ NPs and preserves an active biocompatible cytoskeleton.

## Introduction

Over the past two to three decades, the synthesis and characterization of metal substituted magnetite, Fe_3−x_M_x_O_4_ (M = Mn^2+^, Co^2+^, Zn^2+^, Ni^2+^, Al^3+^, Ga^3+^,etc.) have been intensively researched both for their fundamental scientific interest and for their multiple applications, such as: magnetic storage media^1^, biosensing applications^2^, medical application—targeted drug delivery^3,4^, magnetic hyperthermia^5^, as contrast agents in magnetic resonance imaging (MRI)^6–12^, and magnetic inks for ink jet printing^13^.

These ferrite systems have the cubic ferrite structure and belong to the space group Fd3m. It should be mentioned that the term ferrite is used in a broader sense, which also includes substitutions with trivalent cations. Depending on the metal and cation distribution, the spinel structure can be normal, inversed, and partially inversed^14^. Authors suggested that the properties of these ferrites—magnetic and catalytic—are directly related to the distributions of cations between octahedral and tetrahedral sites in the spinel structure^15,16^. The cation distribution depends on the electronic configuration and ions valence but the changes in the particle size, particularly, at the nanometric scale, can influence and modify the ferrite magnetic behavior^17^. Extensive studies on the magnetic and electric properties have been published, especially on the gallium substitute magnetite, Fe_3−x_Ga_x_O_4_ induced anisotropy. A linear dependence of the Curie temperature versus gallium content has been observed and correlated with the gallium distribution between tetrahedral and octahedral site of the spinel structure. Gamari-Seale et al. studied the magnetite system with low gallium content (x = 0.5 and 0.7)^18^ and pointed out the structural and magnetic of the Fe_1.9_Ga_1.1_O_4_ and FeGa_2_O_4_ systems^18,19^. Later, Dehe et al.^20^ completed the research on the cation distribution in the Fe_3−x_Ga_x_O_4_ spinel system (x ≤ 0.8) using complementary investigation techniques, such as Mössbauer spectroscopy and magnetization measurements.

The technological application of the magnetic ferrite compounds requires the use of targeted properties that can be achieved by controlling the influence of the well-defined processing conditions over the final product structure and morphology. For data storage applications, the magnetic nanoparticles (NPs) need to have a stable and switchable magnetic state to represent bits of information that are not affected by temperature fluctuation^21^.

Chitambar^22^ presented the medical applications and toxicities of gallium compounds, concluding that gallium compounds continue to show promise for the treatment of certain diseases. Recently, it has been suggested that magnetic NPs containing gallium, such as Fe_1.4_Ga_1.6_O_4_ or Mn_x_Ga_1−x_Fe_2_O_4_ (x = 0 ÷ 1), are potential materials for biomedical applications, such as drug delivery systems or hyperthermia treatment^23–25^. Biocompatibility and toxicity are important criteria for biomedical applications, and, in this sense, colloidal stability in water or in different physiological environments of the magnetic NPs is required. Furthermore, the superparamagnetic behavior at room temperature of the monodisperse NPs is preferred. The chemical nature of the magnetically responsive component, the morphology of the particles, such as shapes and sizes, as well as their functionalized surfaces, drastically influence biocompatibility and toxicity^26,27^. The rising prevalence of exposure to nano-iron metal and nano-iron oxides, which are extensively utilized in both engineered and natural settings, has prompted apprehension regarding their potential harm to living organisms due to the generation of reactive oxygen species (ROS) and the subsequent induction of oxidative stress at the cellular level. Substantial research has been conducted to explore the ROS-related behaviors of iron nanostructures in relation to factors such as their chemical composition, particle size, crystalline phase, as well as various bio-microenvironmental factors including physiological pH, buffering agents, biogenic reducing agents, and other organic substances^28,29^.

Another important application of MFe_2_O_4_ (M means metal) NPs is related to the energy transport in superconductors. Here, various ferrites may act as artificial pinning centers which leads to an increase of the critical current value^30^. Recently, there have been reports on the addition of a colloidal solution of the pre-formed CoFe_2_O_4_ and MnFe_2_O_4_ NPs to the YBa_2_Cu_3_O_7−x_ precursor solution used for the growth, by a so-called "ex-situ approach," of epitaxial nanocomposite films through the chemical solution deposition method^31^.

To date, different chemical strategies have been developed to synthesize magnetic ferrite NPs, such as co-precipitation^32,33^, hydrothermal^34^, sol–gel^35^, micro-emulsions^36,37^, or polyol method^13,38^, proving that the physical and chemical properties are strongly dependent on the synthetic route used. Among these methods, the decomposition of metal complexes in a high boiling solvent by solvothermal or microwave-assisted heating methods is preferred because they allow for control of the size and shape of the NPs and their in-situ functionalization, thus preventing their aggregation^38–40^. In comparison with co-precipitation method the thermal decomposition method allows a better control of the surface oxidation process due to the presence of the organic layer on the nanoparticle surface^41^.

A facile and efficient one-pot approach has been thoroughly described by Solano et al.^13^ for both heating up and microwave-assisted decomposition using triethylene glycol (TEG) as the solvent and capping ligand for the MFe_2_O_4_ (M = Mn, Fe, Co, Ni, Zn, and Cu) spinel ferrite magnetic family of NPs. Good control of the morphological characteristics is achieved by adjusting both the temperature ramp and the heat-driven decomposition time^13^. Recently, Sanchez et al. reported the synthesis and characterization of gallium-cobalt ferrites Co_x_Ga_1−x_Fe_2_O_4_ (x = 0.1) NPs with potential use in biomedical applications, using the same thermal decomposition approach, but using tetraethylene glycol as the solvent^25^.

The aim of this study is to synthesize Ga_0.9_Fe_2.1_O_4_ (GaFeO) NPs with good dispersion and crystallinity using two different methods: solvothermal and microwave-assisted heating (MW). The study will then focus on analyzing their structure, morphology, magnetic properties, and biocompatibility. To our knowledge, no reports have been published on the synthesis of nanometric-sized gallium ferrite with this specific stoichiometry of Ga:Fe:O = 0.9:2.1:4. In this study, we obtained concentrated, stable colloidal solutions of GaFeO using TEG as a solvent and capping agent for both methods. By carefully controlling the processing conditions such as temperature, dwell time, heating rate, and decomposition process (solvothermal or microwave), we achieved a narrow size distribution of NPs below 10 nm. We characterized the GaFeO NPs using various techniques including FTIR spectroscopy, thermal analysis (TA), X-ray diffraction (XRD), transmission electron microscopy (TEM), X-ray photoelectron spectroscopy (XPS), magnetic measurements via Superconducting Quantum Interference Device (SQUID), and biocompatibility using human amniotic fluid stem cells (AFSC) through biochemical assay (MTT) and fluorescence microscopy. Our results confirmed the composition and expected spinel structure of the NPs and demonstrated superparamagnetic behavior at room temperature, as well.

## Materials and methods

### Materials

Gallium (III) 2, 4-pentanedionate, GaC_15_H_21_O_6_, IUPAC name: (Z)-4-[[(Z)-4-oxopent-2-en-2-yl]oxy-[(E)-4-oxopent-2-en-2-yl]oxygallanyl]oxypent-3-en-2-one, (Alfa Aesar, 99.99%), iron (III) 2, 4-pentanedionate, FeC_15_H_21_O_6_, IUPAC name: iron(3+) tris((2Z)-4-oxopent-2-en-2-olate), (Alfa Aesar, 99.99%), triethylene glycol, (TEG), C_6_H_14_O_4,_ IUPAC name: 2,2′-[Ethane-1,2-diylbis(oxy)]di(ethan-1-ol), (Alfa Aesar, 99.8%), ethyl acetate, C_4_H_8_O_2_, (Alfa Aesar, 99.8%), ethanol absolute, C_2_H_6_O, (HPLC, Alfa Aesar) were used without further purification.

### Nanoparticle synthesis

*The thermal decomposition approaching*, as described by Cai and Wan^42^ and recently optimized by Solano et al.^13^, was chosen for the synthesis of GaFeO NPs. First, a suspension of Ga(acac)_3_ (0.64 mmol) and Fe(acac)_3_ (1.28 mmol) in 50 mL TEG into an ultrasonic bath was realized. Then the mixture was directly added into a round-bottomed flask equipped with a condenser, a magnetic stirrer, a thermograph, and heating mantle. The system was slowly heated, 1 °C/min, up to 280 °C and kept at this temperature for three different periods of time. According to the dwell time at the reflux temperature, the samples were labeled as GaFeO#1/0 for 0 min, GaFeO#1/1 for 60 min, GaFeO#1/2 for 120 min and GaFeO#1 for 180 min. All samples were cooled at room temperature (ambient rate). Black homogeneous colloidal suspensions containing GaFeO NPs dispersed in TEG were obtained. These colloids are stable at room temperature, even after storage for several months. The GaFeO NPs were separated and washed thoroughly by magnetic precipitation and using a mixture (4:1 vol.) of ethyl acetate and ethanol. Finally, the NPs were dispersed in ethanol forming a stable black dispersion (160 mM).

*The microwave process* started from the same suspension of Ga(acac)_3_ and Fe(acac)_3_ in TEG, as previously described, which was now transferred into a microwave vial. After ultrasonication, the mixture was heated under magnetic stirring by microwave radiation (300 W) up to 220 °C, using a MW oven Discover Explorer Hybrid from CEM. The final temperature was achieved in 10 min with the maximum temperature ramp of 20 °C/min. This temperature was maintained for 10 min then the solution was cooled inside the microwave by the external air flow. The nanocrystals were separated by centrifugation and thoroughly washed with the mixture of ethyl acetate and ethanol thus yielding the GaFeO#2 sample. Redispersion in ethanol produced a suspension which remained stable for several months.

### Characterization techniques

Bright field and High-Resolution Transmission Electron Microscopy images (TEM/HRTEM) coupled with selected area electron diffraction (SAED) were obtained using a 300 kV Tecnai G^2^ F30 S-TWIN transmission electron microscope from FEI. The X-ray diffraction (XRD) measurements were taken using a Bruker D8 Advance diffractometer with Cu X- ray tube and incident beam Ge (111) monocromator (λ = 1.54056 Å). The resulting powders were characterized by thermogravimetric and differential thermal analysis (TG–DTA) using a Mettler Toledo TGA/SDTA851 system, with a platinum crucible, at a heating rate of 10 K/min in air. The chemical nature of the nanoparticle surfaces was analysed by Fourier Transform Infrared Spectroscopy (FT-IR) using a Tensor 27 Bruker FTIR spectrophotometer. The qualitative and quantitative sample compositions were investigated using X-Ray Photoelectron Spectroscopy (XPS) assisted by Ar ions etching. The XPS spectra were recorded using a SPECS spectrometer working with an Al anode (1486.6 eV) as X-rays source. In order to avoid the artificial reduction of the different oxidation stats of elements under the Ar ions beam, the etching was performed by using ions accelerated at a maximum voltage of 1000 V with a filament current of 10 mA. The total content of Fe and Ga was determined through an inductively coupled plasma optical emission spectrometer (ICP-OES) Spectroflame FMD-07 (Spectro Analytical Instruments). The standard solutions used for ICP-OES calibration were purchased form Merck. Ultrapure water (18.2 mΩ cm resistivity) obtained in laboratory with the Millipore equipment (Bedford, USA) was used for dilutions. The magnetization properties were studied using the vibrating sample magnetometer (VSM Lake Shore) and a PPMS 9-QD system (sensitivity 10^–6^ emu, 9-T maximum magnetic field, and temperature range 1.9–1300 K). Magnetization (M) versus applied field (H) was measured for the samples at 4.2 and 300 K. Zero field-cooled (ZFC) and field-cooled (FC) magnetization versus temperature (M-T) measurements were conducted in a 10 Oe magnetic field within the temperature range of 2–400 K.

### Biocompatibility

The study was carried out with the approval of the “Academy of Romanian Scientists “ Ethics Committee, with the approval registration number 778/13.12.2021. The amniotic fluid stem cells used in this research comes from pregnant women’s that test for fetal aneuploidy. After obtaining the results the second cell culture sample used as backup was donate for research upon inform consent of the pregnant women. In this study were used the cells that presents a normal karyotype. All the experiments were carried out in accordance with relevant guidelines and regulations, in accordance with EU regulations and the principles of the Declaration of Helsinki, and upon approval of ethics committee. AFSCs were kindly provided by Genetic Lab S.R.L. diagnoses laboratory, upon written informed consent of the patients, in agreement with national and European Union law. The primary cultures were obtained by centrifugation of amniotic fluid at 1050 rpm for 10 min. The cells were then cultured for 10 days without passages in AmnioMax medium, with medium change every two days (ThermoFischer Scientific, Waltham, Massachusetts, USA). After 10 days, the primary culture was passage and cultured in differentiation specific media supplemented with growth factors. Endothelial differentiation of AFSC was done by culture in M200 medium supplemented with 10% fetal bovine serum (FBS), 40 ng/mL vascular endothelial growth factor (VEGF), 20 ng/mL insulin growth factor (IGF-1), 10 ng/mL epidermal growth factor (EGF), 10 ng/mL basic fibroblast growth factor (bFGF), 100 μg/mL penicillin, 100 μg/mL streptomycin, and 50 μg/ mL neomycin (all purchased from Thermo Fischer Scientific, Waltham, Massachusetts, USA).

The biocompatibility of GaFeO NPs was tested using human amniotic fluid stem cells (AFSC) after 72 h by biochemical assay (MTT assay) and after 5 days by fluorescent microscopy. For the cytotoxicity assessment, MTT assay (CellTiter Non-Radioactive Cell Proliferation Assay, Promega) was employed according to the manufacturer’s guidelines. Briefly, AFSC were grown in 96-well plates, with a seeding density of 3000 cells/well, in different experimental conditions. Then, 15 µL of solution I (3-(4,5-dimethylthiazol-2-yl)-2,5-diphenyltetrazolium bromide) was added and incubated at 37 °C. After 4 h, 100 µL solution II (solubilization solution) was added and pipetted vigorously to solubilize formazan crystals. After 1 h, the absorbance was recorded at 570 nm using a TECAN spectrophotometer. Fluorescent microscopy was performed using RED CMTPX fluorophore (Life Technologies, Invitrogen, USA), a long-term living cell tracker. RED CMTPX dye was added in the culture medium (DMEM medium, Sigma–Aldrich, USA) at a final concentration of 5 mM and incubated for 30 min to allow the dye to penetrate the cells. Afterwards, the cells were washed with phosphate buffered saline (PBS) and visualized by fluorescence microscopy. The photomicrographs were taken with a Carl Zeiss digital camera using Axio-Vision 4.6 software. Cellular organization in the presence of GaFeO#1 and GaFeO#2 was assessed by looking at cytoskeleton tubulin filaments using immunocytochemistry. The AFSCs were cultured and then twice washed with PBS. They were fixed for 20 min with 4% paraformaldehyde in PBS, rewashed two times with PBS, permeabilized with 0.3% Triton X-100 in PBS (PBS-T) for 2 × 15 min, blocked with 4% normal goat serum in PBS-T (which was used for all subsequent washing and dilution of antibodies) for 1 h, treated with primary antibodies (tubulin, mouse monoclonal, 1:4000) versus negative control (dilution vehicle only) for 3 h at room temperature, washed 3 × 2–3 min, treated with secondary antibody (AlexaFluor 488 goat anti-mouse, Invitrogen, 1:1000) for 1 h at room temperature, washed 3 × 2–3 min and 2 × 1 min with purified water, mounted on glass microscopy slides with ProLong Gold anti fade with DAPI (Invitrogen P36935), and examined with an inverted fluorescence microscope.

## Results and discussion

Today it is generally accepted that the formation mechanism of metal oxide NPs by wet-chemical methods consists in two main stages as described by LaMer’s model: a nucleation step followed by the particle’s growth step^43^. LaMer et al. described the formation of seeds by increasing the precursor monomer concentration up to the supersaturation level. The NPs are generated by a depletion of the monomer concentration as a result of their continuous aggregation onto the seeds. The chemical nature of the solvent influences both the nucleation and growth processes. This can be explained by the stability of the metal organic complexes that depends on the functional group of the solvent^42–44^. According to Solano et al. the use of TEG as reaction medium induces a limiting effect for the growth of MFe_2_O_4_ NPs during heating stage (150 min)^13^. In this vein, our synthesis has been conducted using TEG as reaction medium for both approaches. To study the influence of the reaction time at the reflux temperature for the solvothermal approach, the synthesis has been done in standard conditions but up to a reaction time of 180 min.

### Morphological characterization

The TEM and HRTEM images for the NPs synthesized by solvothermal approach at different reaction times from 0 to 1.5 h are presented in Fig. S1 in the Supplemental data. When the reaction mixture reaches the TEG boiling temperature (T = 280 °C, t = 0 h), the HRTEM image suggests that the nucleation step just occurred. After reflux for 1 h, the formation of NPs can be noticed, although they are not homogeneous in size.

**Figure 1:**
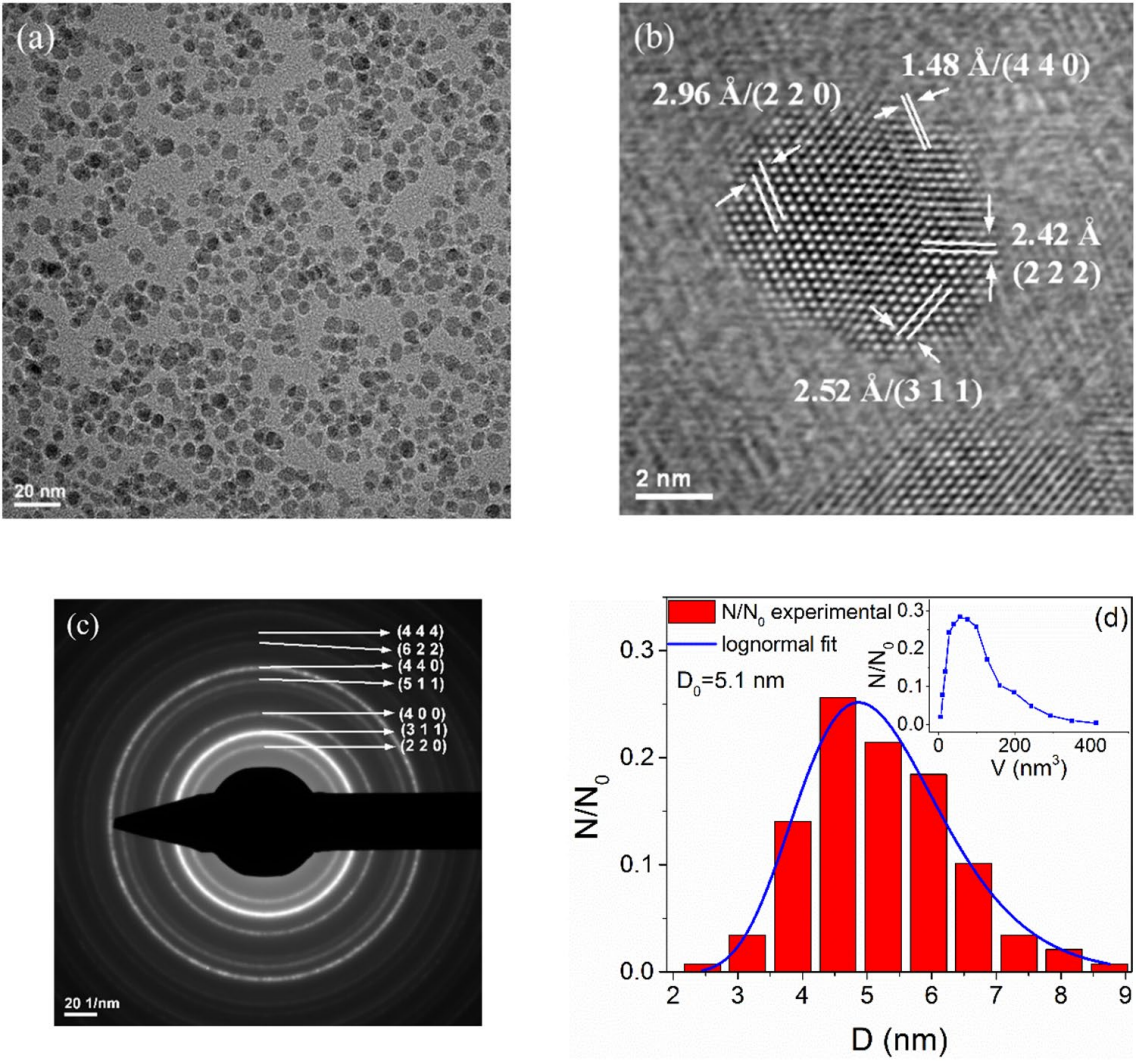
(**a**) TEM, (**b**) HRTEM images, and (**c**) SAED pattern from GaFeO#1 sample. (**d**) Normalized diameter distribution with, as inset, the corresponding normalized volume distribution.

The TEM and HRTEM images, SAED patterns, and particle diameter distribution for the GaFeO#1 sample (3 h) are presented in Fig. 1a-c. The GaFeO NPs present rounded shapes, low aggregation, and an average diameter of 5.1 nm. The HRTEM image in Fig. 1b shows various crystalline planes according to the orientation of this nanoparticle with respect to the direction of the electron beam. The following interplanar distances d_hkl_ have been directly observed: 0.296, 0.252, 0.242, and 0.148 nm, which correspond to the (220), (311), (222), and (440) plane families of the spinel structure, respectively. The electron diffraction patterns present rings indexed with hkl reflections of Ga_0.9_Fe_2.1_O_4_ spinel structure, as indicated in Fig. 1c, thus confirming the formation of well-crystallized NPs.

**Figure 2:**
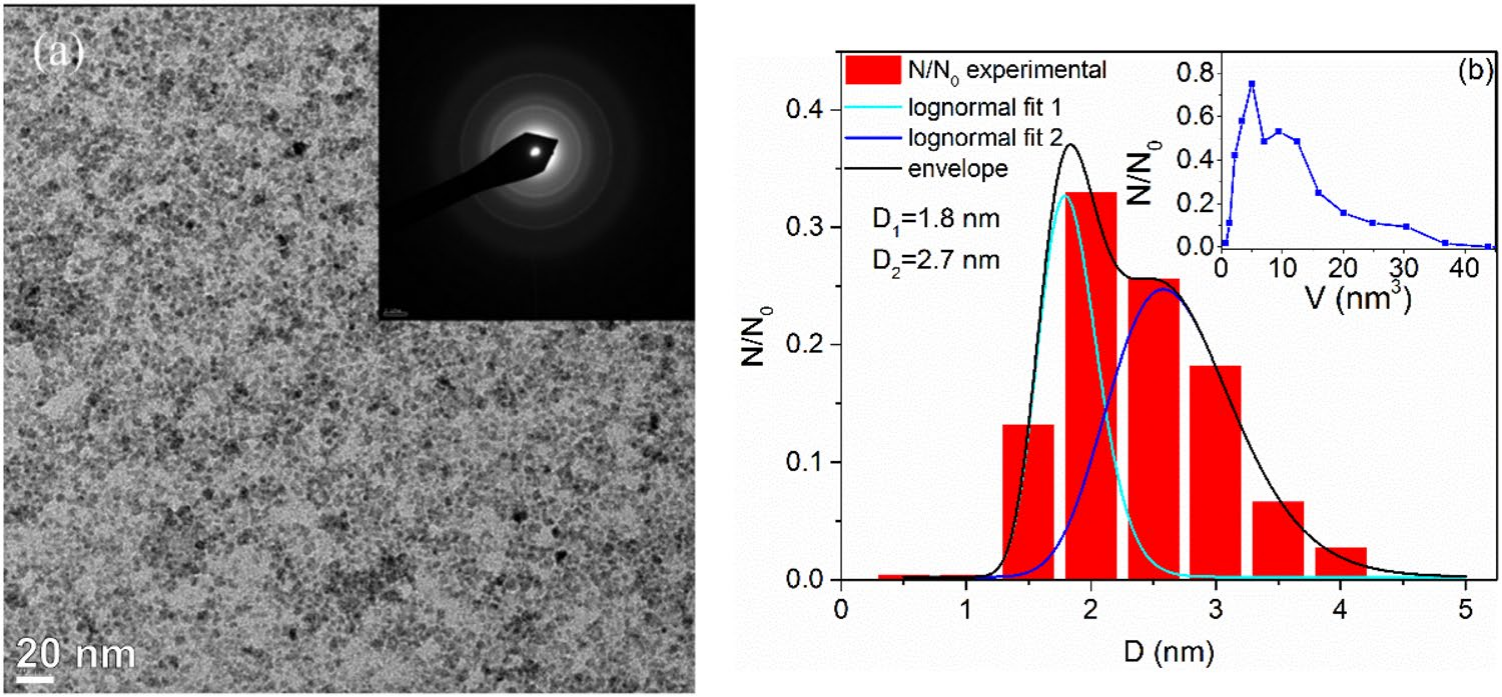
(**a**) TEM image and SAED patterns (inset) for GaFeO#2 sample and (**b**) the bimodal normalized diameter distribution with the corresponding normalized volume distribution in the inset.

In the case of the GaFeO#2 sample, the same shapes and low aggregation of the NPs can be observed. The image is shown in Fig. 2a. The inset presents the corresponding diffraction patterns, but their indexation cannot be realized in a simple manner. Figures 1d and 2b show the normalized diameter distributions for the two samples. The corresponding insets represent the normalized volume distributions. A bimodal distribution can be noticed in the case of the microwave-prepared sample, GaFeO#2, with the maxima at 1.8 and 2.7 nm. For both samples the diameters of at least 400 NPs were determined for the statistics. The number and sizes of bins was determined by using the Sturges criterion.

### Structural characterization

**Figure 3:**
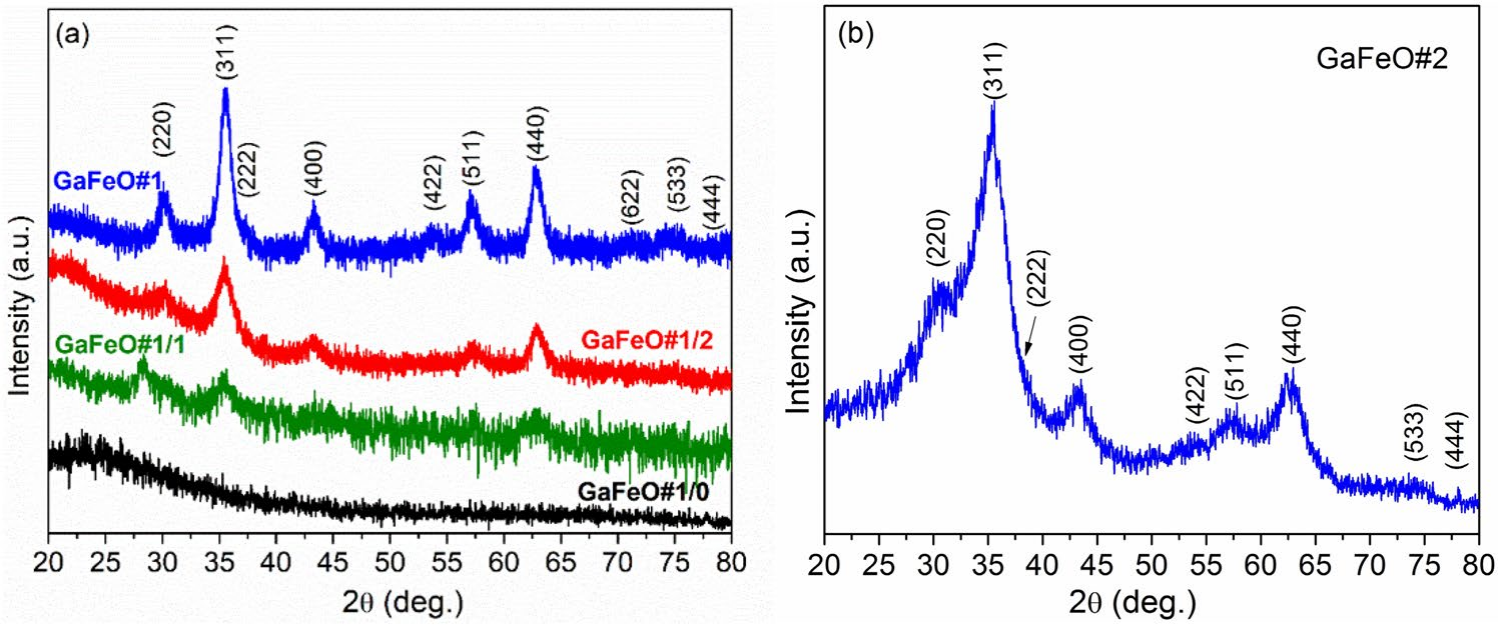
XRD patterns of the GaFeO NPs obtained by (**a**) solvothermal and (**b**) microwave assisted routes. The reflections have been indexed according to the JCDD PDF 074-2226 for Ga_0.9_Fe_2.1_O_4_ spinel structure. (GaFeO#1/0, GaFeO#1/1, and GaFeO#1/2 dwell time 0, 1, and 2 h).

The crystalline structure of the GaFeO NPs was studied using X-ray diffraction (XRD), and the corresponding diffraction peaks are shown in Fig. 3a,b. All the reflections could be indexed to the spinel structure of Ga_0.9_Fe_2.1_O_4_ (JCDD PDF 074-2226). Additional peaks representing supplementary phases are not observed here.

To study the influence of reaction time on the nucleation and growth of the NPs, different solvothermal syntheses were performed at various dwell times. Figure 3 shows the diffraction patterns of the reaction products corresponding to different dwell times. After a reflux of 1 h, as for GaFeO#1/1 sample, the Ga_0.9_Fe_2.1_O_4_ diffraction patterns (spinel) show low-intensity Bragg reflections. It is an indication that nucleation has already started, and the nanoparticle growth process is ongoing. At longer reflux times (GaFeO#1/2 sample), grain growth continues, as illustrated by the increase in intensity of all Bragg reflections. After 3 h, as indicated by the diffraction patterns of the sample labeled GaFeO#1, the spinel structure is fully formed.

The average particle size of the fully synthesized sample was calculated using the Scherrer method and the (311), (440), (511) reflection peaks, resulting in a mean value of approximately 5.1 nm. The value calculated from the XRD data is the same as the mean particle size value obtained from TEM images (Fig. 1d). This indicates that the particles are mainly formed by nanocrystallites.

On the other hand, the XRD peak broadenings observed in the case of the GaFeO#2 sample indicate smaller dimensions than for the GaFeO#1 sample. Here, the calculated value through the Scherrer method is around 2.3 nm. It should be mentioned that these very broad peaks, as shown in Fig. 3b, have an uncertain baseline with a generally decreasing slope at high 2θ values. This is an indication that, besides the crystalline part, a large amount of amorphous material is contained within this sample. To further check the above-mentioned situation the Rietveld refinement was conducted by using the mentioned PDF 074-2226 file for Ga_0.9_Fe_2.1_O_4_ spinel structure. The convergence was achieved only for GaFeO#1 sample where the amorphous phase is minimal. The resulted fit is shown in Fig. S2 from the Supplemental data file. In case of this sample, all the determined lattice parameters and angles indicate the formation of the undistorted cubic spinel structure. The determined mean crystallite dimension was found to be around 5.7(4) nm, in accordance with its other evaluations.

### Compositional characterization

**Figure 4:**
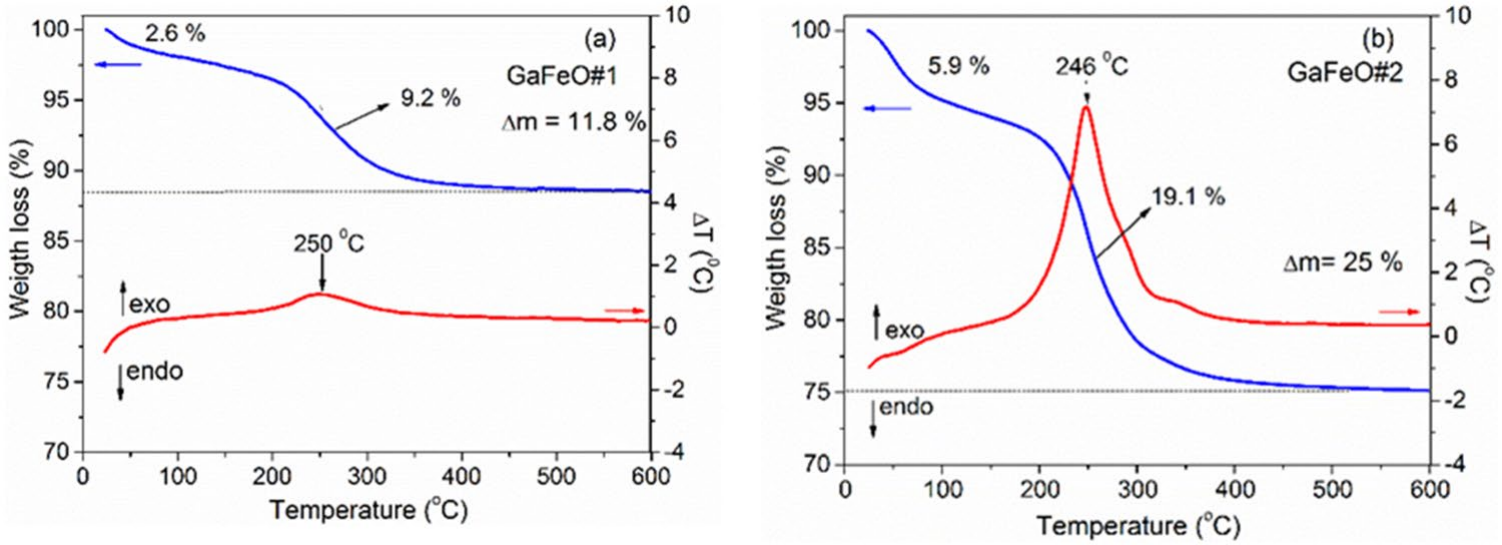
TG–DTA analysis of the GaFeO NPs: (**a**) GaFeO#1sample prepared by the solvothermal and (**b**) GaFeO#2 sample prepared by microwave assisted decomposition.

The presence of polyol ligands on the surface of the GaFeO NPs, as a residue resulting from the preparation routes of the two samples, is evidenced by both TG–DTA and FTIR measurements. Figure 4 shows the TGA and DTA curves for GaFeO #1 and #2 NPs. The thermal analyses reveal two successive stages of weight loss: one in the temperature ranges of 25–200 °C, and the other between 200 and 350 °C. The first stage, with mass losses of 2.5 wt% for GaFeO#1 and 5.9 wt% for GaFeO#2, is attributed to the elimination of physically adsorbed solvents, such as ethanol or ethyl acetate. For both samples, the second stage represents the most significant weight loss, namely 9.1% for GaFeO#1 and 19.1% for GaFeO#2. It is attributed to the exothermic removal of residual TEG from the nanoparticle surfaces. In correlation with its smaller nanoparticle sizes and high specific surface area, both thermal processes are enhanced for the GaFeO#2 sample.

### FT-IR spectroscopy

**Figure 5:**
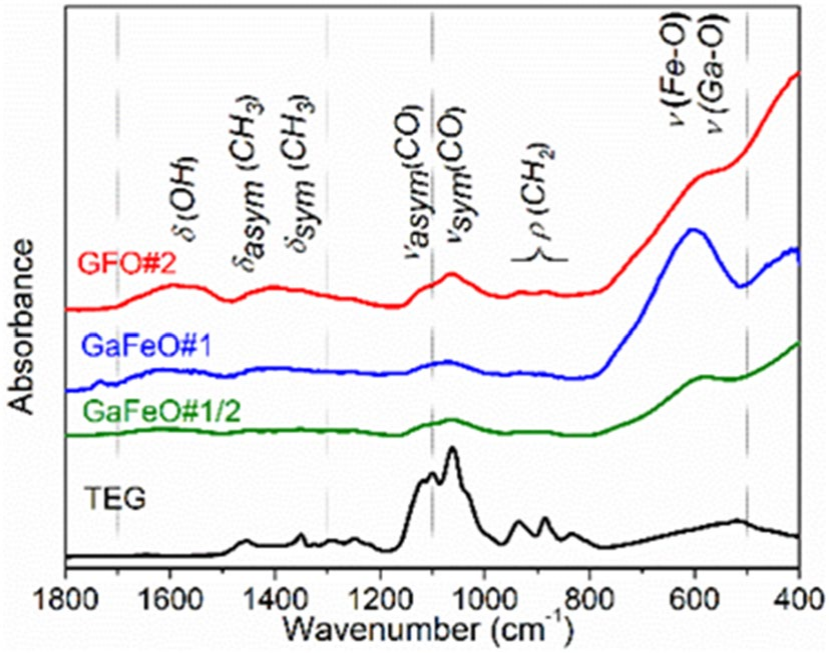
FT-IR spectra of TEG and GaFeO samples.

In order to understand and analyze the chemical nature of the organic species stabilizing the NPs, FT-IR spectroscopy was used. The results are presented in Fig. 5. The presence of TEG molecules on the surface of the NPs is confirmed through the characteristic strong absorption peaks at 1050–1100 cm^−1^ representing the symmetric and asymmetric stretching modes (ν_sym_ and ν_asym_) specific to the C–O–C bonds. It should be mentioned that these organic residual groups are either covalently bonded or chemisorbed onto the particle surfaces where they form a protective shell^45^. The FT-IR spectra also present high intensity bands at 764, 620, 598 and 443 cm^−1^ which can be attributed to Fe–O and Ga–O metal–oxygen interactions^46,47^.

### The XPS analysis of samples

**Figure 6:**
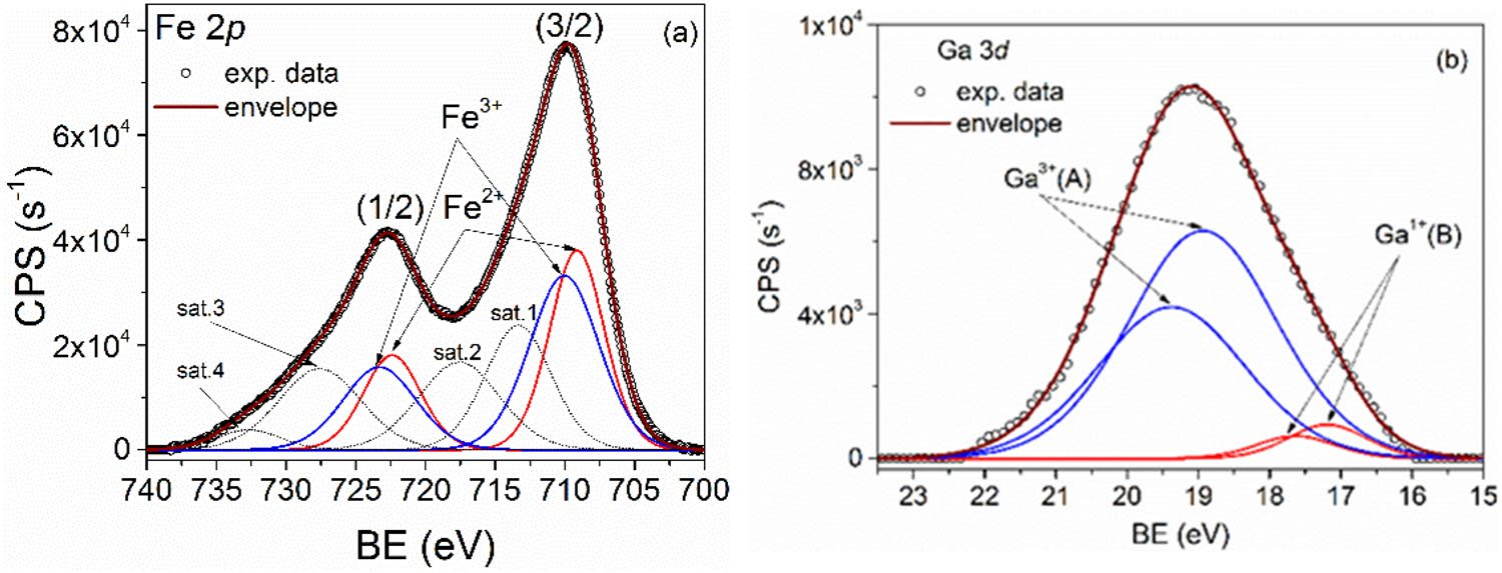
XPS spectra of (**a**) Fe (2p), (**b**) Ga (3d) core-levels together with the corresponding deconvolutions for the GaFeO#1 sample.

**Figure 7:**
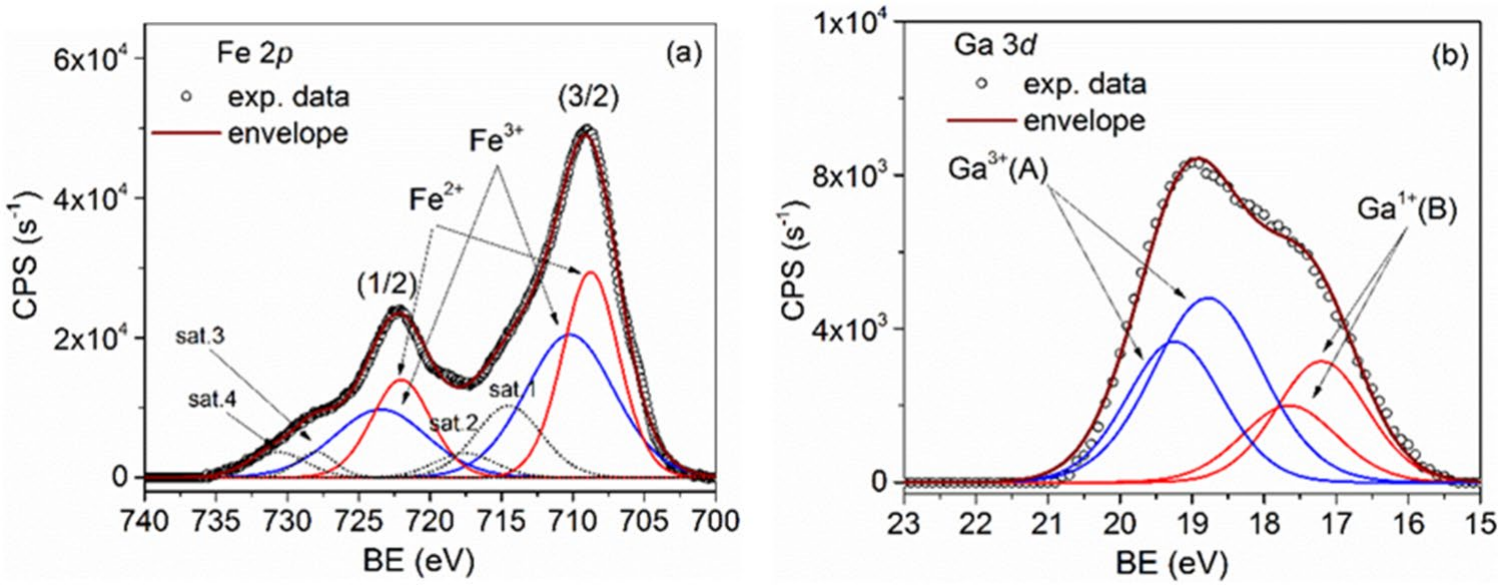
XPS spectra of (**a**) Fe (2p), (**b**) Ga (3d) core-levels together with the corresponding deconvolutions for the GaFeO#2 sample.

The sample compositions were determined by the XPS technique. The XPS recorded spectra of Fe 2p core-level doublet recorded for the GaFeO#1 and GaFe#2 samples, together with the corresponding deconvolutions and the fitted envelope, are shown in Figs. 6a and 7a respectively. For both 2p (3/2) and 2p (1/2) core-level lines the deconvolutions were realized by considering the Fe^2+^ and Fe^3+^ states as components^48^. The low energy peaks (labels A) belong to Fe^2+^ while the higher ones (labels B) belong to the more oxidized Fe^3+^ state. At higher energies, for each Fe oxidation state two shake-up satellite features appear in spectra around 713.3 and 717.5 eV for (3/2) peaks and at 727.6 and 732.6 eV for (1/2) peaks, respectively^48^. The restrictions used to fit the Fe 2*p* XPS spectra refer to the relation between areas of the two components, A_1/2_ = A_3/2_/2, and to the spin–orbit doublet energy separation which was adjusted between 13.2 and 13.4 eV. The ratios between (1/2) and (3/2) linewidths were set between 1 and 1.1.

For the analysis of the gallium presence in samples the Ga 3*d* core-level was chosen because it is more sensitive to chemical shifts while the Ga 2*p* core-level, even if more intense, shows very small chemical shifts. The deconvoluted spectra of Ga 3*d* core-level lines are presented in Figs. 6b and 7b, for the GaFeO#1 and GaFeO#2 samples, respectively. In these figures, besides Ga 3*d* doublet lines (labeled A), associated to Ga_0.9_Fe_2.1_O_4_, and positioned at 18.93 (5/2) and 19.37 (3/2) eV, another Ga 3*d* doublet (labeled B), was evidenced at 17.19 (5/2) and 17.63(3/2) eV respectively. The latter doublet arises from Ga^+^ ions belonging to some Ga_2_O sub-oxide phase. The spin–orbit splitting of Ga 3*d* core-level lines was set at 0.44 eV for both quantifications.

To summarize, both samples contain [Fe^2+^O]^T^[Ga^3+^_0.9_Fe^3+^_1.1_O_3_]^O^ and Ga^1+^_2_O secondary sub-oxide phase, but in different relative concentrations. As it can be seen in Fig. 7b a larger quantity of Ga sub-oxide is formed in case of microwave synthesis route (GaFeO#2) than in the solvothermal preparation of GaFeO#1 sample (Fig. 6b).

The analysis of C 1*s* spectra is in accordance with the expected residual carbon and carbon compounds as found by TG and DTG and other complementary methods^38^. The C 1*s* core-level spectra are shown in Figs. S3 and S4 from the Supplemental data material. For XPS spectra calibration of both samples the C–C/CH peaks were positioned at a BE of 284.6 eV. The presence of low intensity C 1*s* peaks at BE greater than of 288 eV corresponds to the presence of different organic residues.

The higher decomposition rate of Ga(acac)_3_ relative to Fe(acac)_3_^45,49^ causes an unbalance in the formation of compounds with different stoichiometry. In case of GaFeO#1 the solvothermal reaction at 280 °C is almost completed and the residual compounds are only formed by small amounts of gallium sub-oxide Ga_2_O, TEG chains and residual carbon or C-H moieties anchored on the particle surfaces. In general, XPS is a very useful technique in the compositional determination of surfaces. In the case of nanostructured materials that can contain several phases, through this method, when associated with Ar ions etching, complex quantitative compositional determinations can be obtained^50^. For the compositional analysis all the integral intensities were calibrated by using the relative sensitivities, transmission and electronic mean free path factors as given in CASA software database. Each of the two samples were subject to consecutive etchings with Ar ions until the shape and intensity of XPS spectra remained unchanged. At this stage, the quantitative composition of samples can be calculated by taking into account the escape depths of each identified component^50–52^. To account this, the calculated integral intensities were divided to the corresponding escape depths expressed in nm. Following Refs.^50,53,54^ the calculated escape depths are as following: 0.88 nm for Ga_0.9_Fe_2.1_O_4_ (Fe 2p_3/2_ kinetic energy—KE), 1.84 nm for Ga_2_O (Ga 3d 5/2 KE), 2.64 nm for C_6_H_14_O_4_ (C 1s KE) and 2.24 nm in case of residual carbon (C 1s KE). Line positions and normalized integral intensities are presented in Tables S1 and S2 from the Supplemental data. Here the Table 1 summarizes the compositional analysis of GaFeO samples. In order to check the XPS calculated compositions the results from ICP-AES determinations are also presented here.

**Table 1:** The compositional analysis of GaFeO samples.

Sample	Ga_0.9_Fe_2.1_O_4_ (wt%)	Ga_2_O (wt%)	C_6_H_14_O_4_ (wt%)	Carbon residues (wt%)	Ga:Fe (mass ratio)	
					ICP-AES	XPS
GaFeO#1	95	2	2	1	0.56	0.57
GaFeO#2	**85.1**	6.8	5.7	2.4	0.78	0.81

### Magnetic characterization

**Figure 8:**
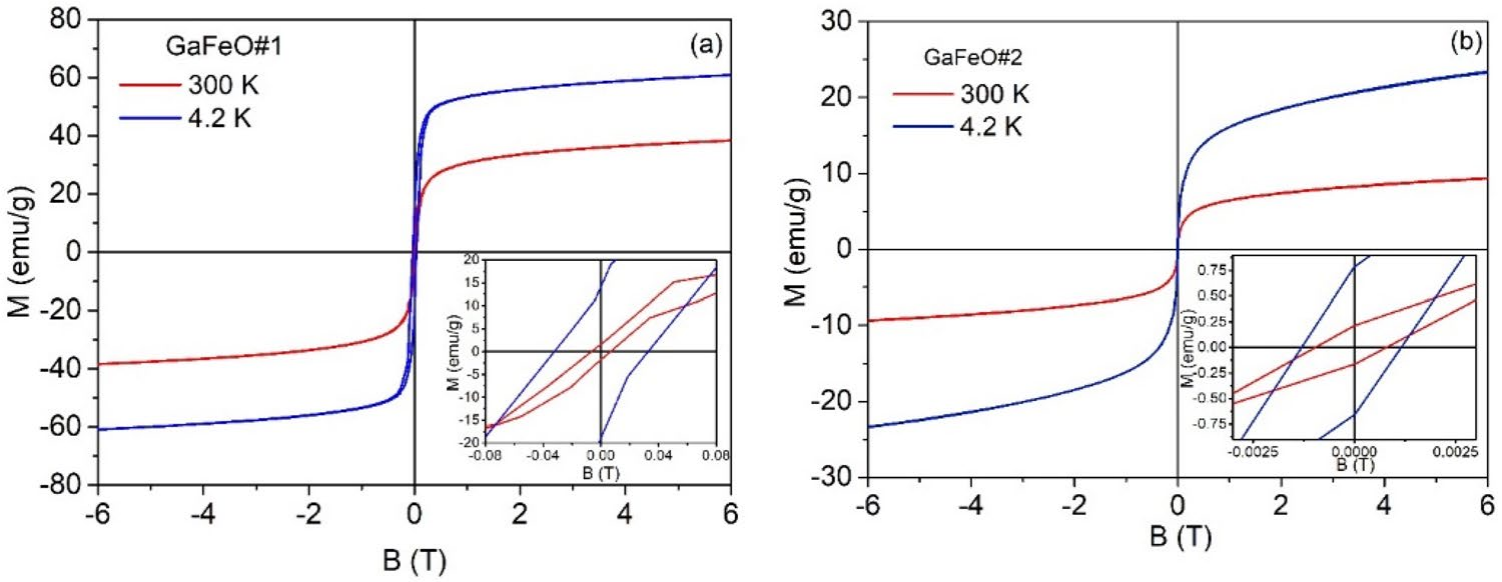
Magnetization of samples (**a**) GaFeO#1 and (**b**) GaFeO#2 as a function of the applied magnetic field, determined at room temperature and 4.2 K.

The magnetic properties of the NPs were determined using the superconducting quantum interface device (SQUID) magnetometry. Figure 8a,b show the magnetization behavior of samples GaFeO#1 and GaFeO#2 as a function of the applied magnetic field, respectively. The values of the saturation magnetization (M_S_) and coercivity field (H_C_) for sample GaFeO#1, at 300 K, are 43.88 emu/g and 65 Oe while, at 4.2 K, the corresponding values are found to be 66.5 emu/g and 327 Oe respectively. The ratio M_S_/M_R_, with M_R_ representing the remanent magnetization, was calculated as 0.03 at 300 K and 0.25 at 4.2 K for the GaFeO#1 sample, as it can be seen from the inset of Fig. 8a. In contrast, for the GaFeO#2 sample, the values were 0.02 at 300 K and 0.03 at 4.2 K (inset of Fig. 8b). These values, along with the low coercive fields observed in both samples, suggest that the nanoparticle ensembles exhibit superparamagnetic behaviour within this temperature range. In the case of the GaFeO#2 sample, the very small variation, with respect to the temperature, of the M_R_/M_S_ ratio should be noted. The saturation magnetizations were calculated in the limit as 1/B approaches 0. The low values of the saturation magnetization indicate that in the case of the GaFeO#2 sample, there is a rather large number of nanoparticles without magnetic ordering associated with their poor crystallinity. These small particles have surface non-correlated magnetic moments, in accordance with the analysis of the XRD patterns. When expressed in Bohr magnetons per formula unit (μ_B_/f.u.), the magnetizations of the GaFeO#1 sample are 2.02 μ_B_/f.u. at 300 K and 3.06 μ_B_/f.u. at 4.2 K. For the GaFeO#2 sample, the corresponding values are 0.5 and 0.6 μ_B_/f.u. at 300 K and 4.2 K, respectively. In calculating these values, the paramagnetic contributions were extracted. For the two samples, the corresponding concentrations of material showing magnetic ordering were considered, as indicated in Table 1.

On the origin of these values, especially in the case of sample 1, the following qualitative considerations can be made: (i) The arrangement of cations in the spinel structure indicates a stoichiometry of 0.9 ions per formula unit (f.u.) of Ga^3+^ and 1.1 ions per f.u. of Fe^3+^ in the octahedral positions, while the tetrahedral positions are exclusively occupied by Fe^2+^ ions (1 ion per f.u.). (ii) Due to the presence of Ga ions, assumed to be uniformly distributed in the network within the octahedral positions, the overlap of the 3d^5^ wave functions of the Fe^3+^ ions with oxygen and gallium is very limited, thus favoring their antiferromagnetic ordering. (iii) In this case, the observed magnetization can only arise from the ferromagnetic coupling of Fe^2+^ ions (3d^6^ in the high-spin configuration) in tetrahedral positions, facilitated by superexchange interactions mediated by oxygen ions. According to this hypothesis, the maximum number of Bohr magnetons is 4 μ_B_, which corresponds to the value of 3.06 μ_B_ obtained at low temperatures. The difference is attributed to surface states, where the magnetic moments are likely canted into a glassed state.

The magnetization field dependence of an ensemble of superparamagnetic particles can be effectively described using the following equation:

1 $$\mathrm{M}\left(\mathrm{H},\mathrm{T}\right)={\mathrm{M}}_{\mathrm{s}}\frac{\int \mathrm{V}\left(\mathrm{D}\right)\mathrm{L}\left[{\mathrm{M}}_{\mathrm{s}}\mathrm{V}\left(\mathrm{D}\right)\mathrm{H}/{\mathrm{K}}_{\mathrm{B}}\mathrm{T}\right]\mathrm{f}\left(\mathrm{D}\right)\mathrm{dD}}{\int \mathrm{V}\left(\mathrm{D}\right)\mathrm{f}\left(\mathrm{D}\right)\mathrm{dD}}$$

here: V(D) represents the volume of the magnetically ordered nanoparticles, which is expressed as a function of their diameters, H stands for the applied external magnetic field, L is Langevin's function, M_s_ represents the saturation magnetization, which is normalized to the magnetic content of the samples, as detailed in Table 1. Also, f(D) is the normalized distribution of magnetic diameters, and it is described by a lognormal distribution function that includes the mean diameter D_0_ and its dispersion σ. The fitting process was carried out numerically using our FORTRAN application, with D_0_, M_s_, and σ as the fitting parameters, as referenced in Refs.^55–60^. The paramagnetic contribution was first extracted before fitting, and then it was reintroduced.

**Figure 9:**
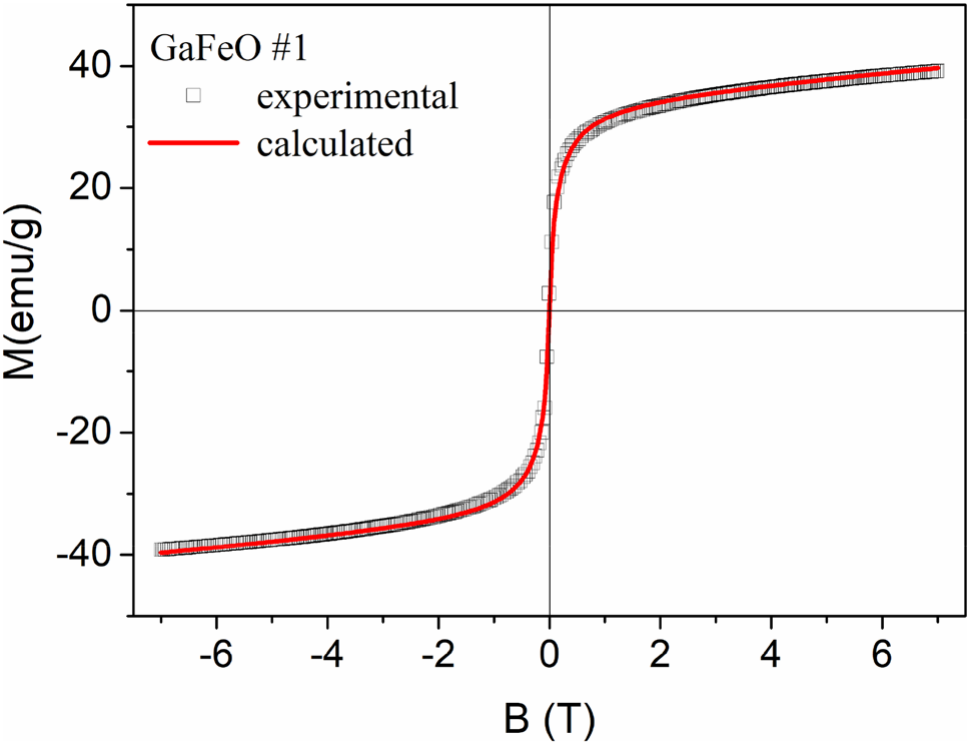
The magnetization curve of the GaFeO#1 NPs at room temperature (□) together with the best fit obtained by using Eq. 1 (continuous line). The magnetizations were calculated referred to the magnetic content of this sample as given in Table 1.

Thus, for GaFeO #1 sample the field dependence of the magnetization, *M* = *f(H)*, is represented in Fig. 9 by a continuous line. It is superposed to the experimental demagnetizing data. The fit resulting parameter values are: *D*_*0*_ = 5.1 nm, M_S_ = 45.1 emu/g, and σ = 0.38. It can be seen that D_*0*_ value is very close to value derived from the TEM data while σ is slightly larger than the value resulted from the statistical analysis of TEM images (Fig. 1a). In the case of sample GaFeO#2, achieving a proper fit was only possible by reducing the mass concentration of magnetic material within the composite to a range of approximately 0.4–0.6%. This reduction means that, out of the total composition of approximately 0.85%, slightly more than half of the nanoparticles possess cores with magnetic ordering capable of superparamagnetic rotation. This difference is likely attributed to the presence of very small nanoparticles, measuring below 2 nm. Even if these nanoparticles maintain the correct stoichiometric composition, their magnetic moments become frozen due to surface effects, resulting in a minimal contribution of this nanoparticle sub-assembly to the overall magnetization. As mentioned, within this procedure a linear paramagnetic term was extracted from the reordered data before fitting procedure. This alignment between experimental and calculated curves is illustrated in Fig. S5. Here, the determined values for M_s_, D_0_ and σ are 8.0 emu/g, 2.5 nm and 0.65, respectively for a GaFeO#2 mass concentration of 0.5%. However, these values are not correct since they don’t reflect the *entire* behaviour of the ensemble.

Additionally, an alternative set of solutions can be derived at higher concentrations, approximately 0.75%. Under these conditions, the calculated magnetic core diameters fall below 1 nm. This scenario represents the point where the model's practical relevance is maximally challenged. It's worth noting that NPs exhibit a core–shell structure in terms of magnetic moments. At extremely small external dimensions, the magnetically ordered core essentially loses its significance, as it is overshadowed by the frozen magnetic moments within the outer shell. Additional details are given in the Supplemental. Thus, the number of parameters becomes too large, and the fitting process gave multiple solutions, mostly unphysical.

**Figure 10:**
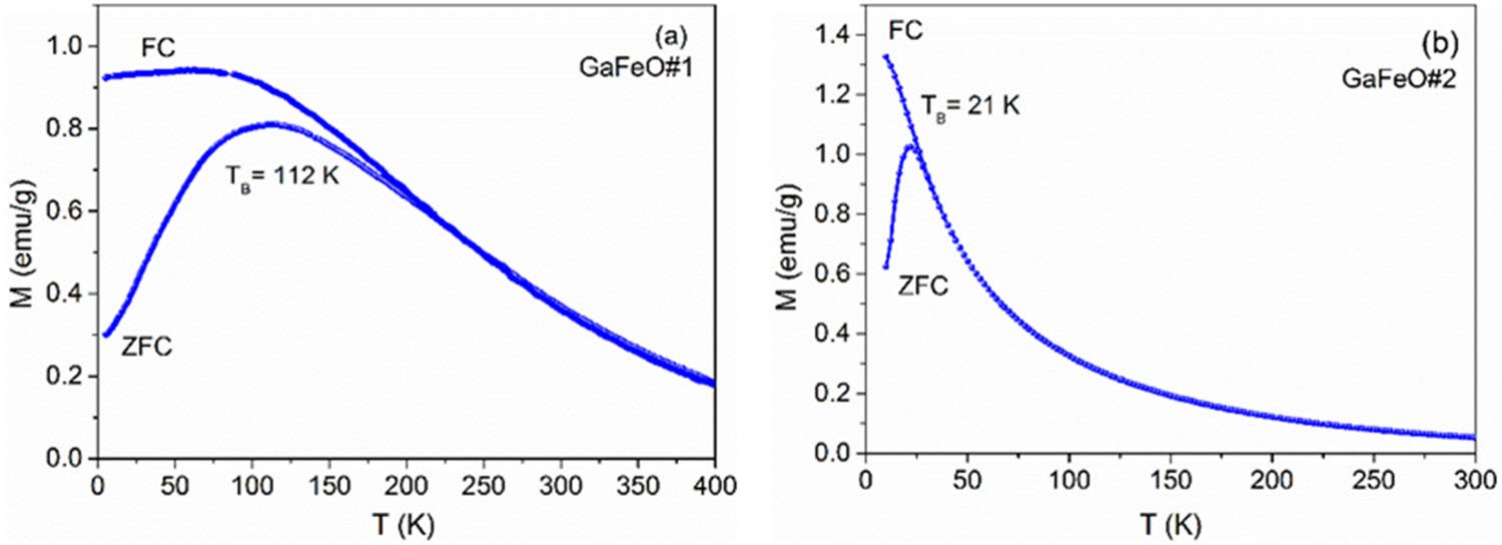
FC and ZFC curves highlighting changes in magnetization of NPs as a function of temperature for (**a**) GaFeO#1 and (**b**) GaFeO#2 samples.

Field-cooled (FC) and zero-field-cooled (ZFC) measurements are presented in Fig. 10a for GaFeO#1 and 10(b) for GaFeO#2. The results support the assumption of superparamagnetic behavior in the samples. The ZFC magnetization increases with temperature until it reaches a maximum value at the blocking temperature (TB). Above this temperature, thermal energy surpasses the magnetic anisotropy energy barrier, ΔE, causing the nanoparticles to exhibit superparamagnetic behavior^61^.

The spontaneous temperature evolution of the magnetization of an ensemble of superparamagnetic nanoparticles, during heating in a zero magnetic field and after a field cooling process, represents the so-called thermoremanent magnetization (TRM). TRM is a measure of the sequential deblocking process occurring within the ensemble of nanoparticles, each with a specific volume distribution. It can be measured directly or determined by the difference between the temperature dependencies of the magnetization curves for field cooling (FC) and zero-field cooling (ZFC)^62^. The first derivative of TRM provides insight into the distribution of energy barriers, denoted as ΔE, which must be overcome during the thermal deblocking of magnetic moments^63^. For of a monodomain NP of volume V the height of the energy barrier, is given by the relation $\Delta E={K}_{eff}V= {k}_{B}T ln\frac{{t}_{m}}{{\tau }_{0}}$. Here *K*_*eff*_ is the effective anisotropy energy density, *T* is the temperature, *k*_B_ is the Boltzmann constant, τ_0_ represents a microscopic relaxation time, and *t*_*m*_ is a relaxation time seen as the measurement time. Since τ_0_ is of the order of 10^–9^ s and *t*_*m*_ has values between 10 and 100 s the value of the logarithm is between 23 and 27^62,64^.

**Figure 11:**
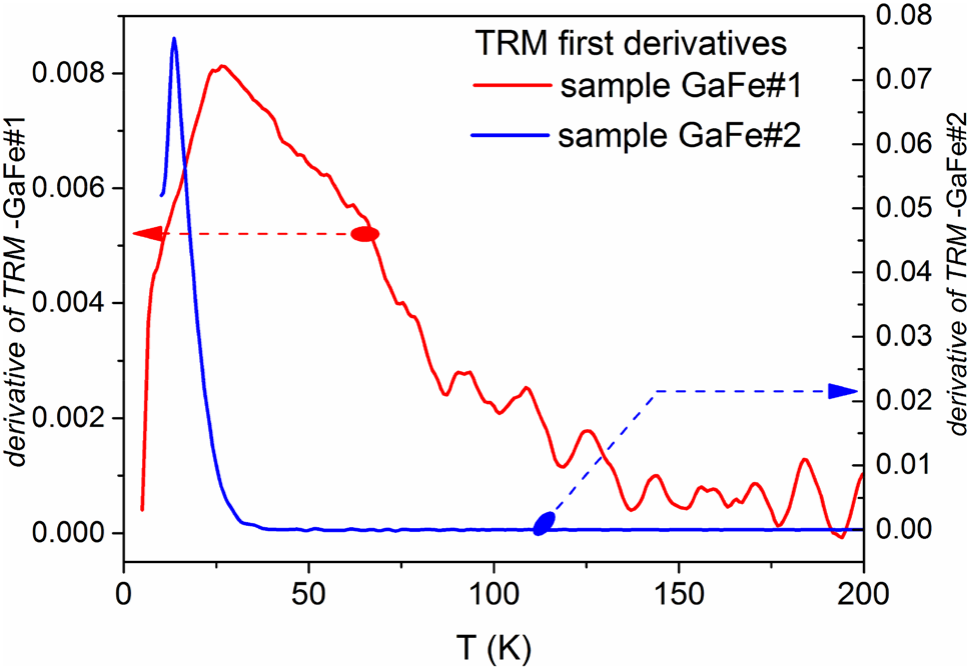
First derivative of TRM magnetizations for samples GaFeO#1 and GaFeO#2.

The first derivatives of TRM functions are presented in Fig. 11 for both GaFeO#1 and GaFeO#2 samples. The discrepancy between the two samples is obvious and it is due to their different magnetic behaviors. Considering a value of 25 for $ln\frac{{t}_{m}}{{\tau }_{0}}$ the values of the axial effective anisotropy constant *K*_*eff*_ were calculated^62^ as 1.38 × 10^5^ and 9.1 × 10^5^ J/m^3^ for GaFeO#1 and GaFeO#2 samples, respectively. Supposing that these values are slowly depending on the deblocking temperatures of various volume fractions from a given nanoparticle ensemble, a reconstruction of the volume distributions can be done starting from the energy barriers distributions given in Fig. 11.

**Figure 12:**
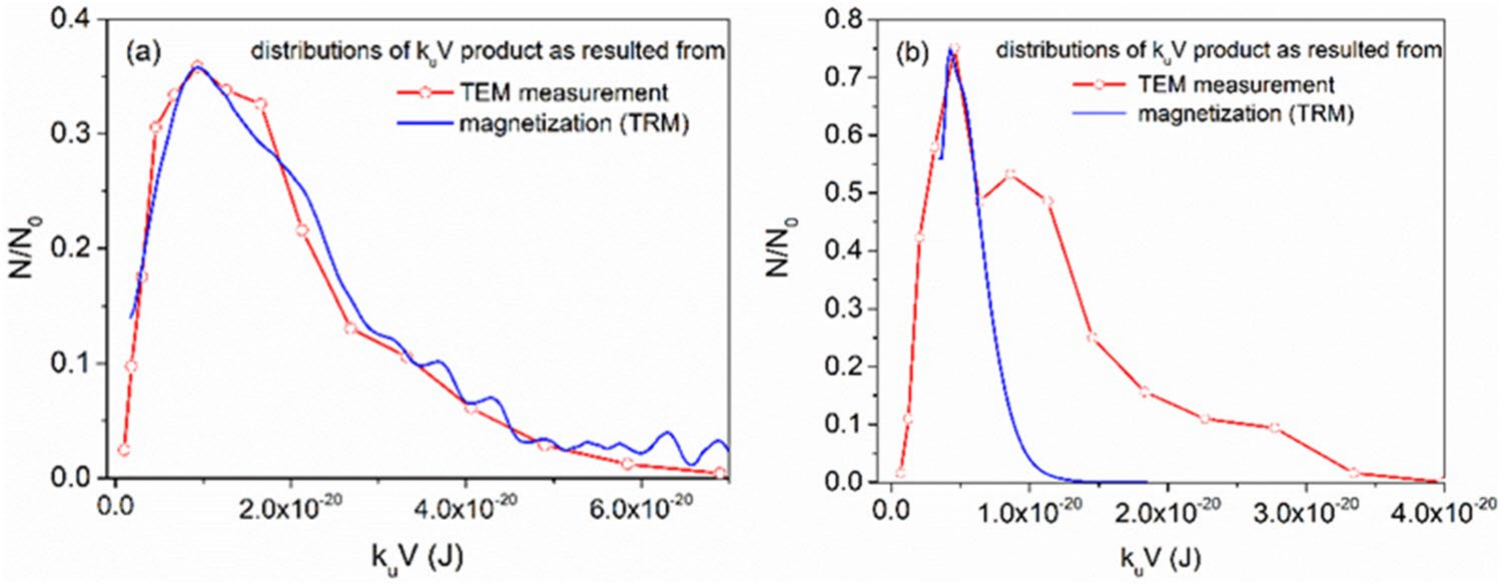
Compared distributions of the ${\mathrm{k}}_{eff}\mathrm{V}$ product as resulted from TEM and magnetic measurements for (**a**) GaFeO#1 and (**b**) GaFeO#2 samples.

The same distribution of the heights of the energy barriers can be reconstructed from the diameter measurements in the TEM images. For comparison, in Fig. 12a,b are represented the superposed energy barriers distributions, $\Delta E={k}_{eff}V$, reconstructed from both TRM and TEM data for GaFeO#1 and GaFeO#2 samples respectively.

As can be seen from Fig. 12a, in the case of sample GaFeO#1, the overlap of the two distributions is very good, which means that, practically, the entire set of NPs in the mentioned sample is represented by crystallites, each of them having ferromagnetic ordering. Regarding GaFeO#2 sample, as shown in Fig. 12b, the superposition appears only for the peak positioned at low energy values of the ${k}_{eff}V$ product. It is evident that several types of nanoparticles (NPs) are present within the sample, resulting in a bimodal distribution: NPs with small dimensions exhibit ferromagnetic ordering (as indicated by the blue curve), while the even smaller ones possess spin-canted states (especially for GFO#2 sample). Additionally, a significant portion consists of amorphous NPs with slightly larger dimensions, lacking magnetic ordering. These findings align with the results obtained from XRD and SAED. Consequently, this approach has allowed us to test the validity and limitations of the superparamagnetic model.

### Toxicity studies

**Figure 13:**
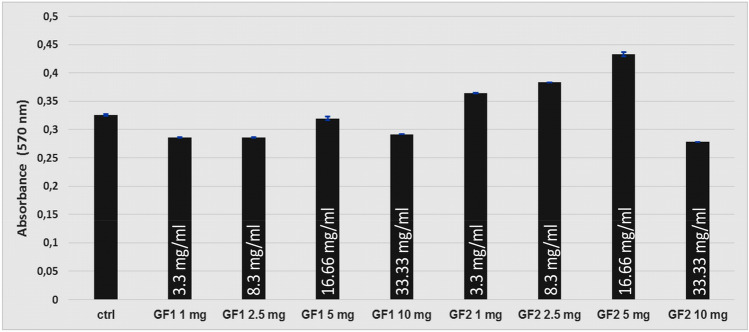
The measured values of the absorbance at 570 nm of the AFSC after 72 h of incubation in the presence of different quantities of GaFeO NPs as compared to the control cells. Here GF1 and GF2 means GaFeO#1 and GaFeO#2 samples respectively.

Data from stem cell studies suggest that amniotic fluid cells are a rich source of stem cells that could be used in tissue engineering for various applications in regenerative medicine^65^. AFSCs are a heterogeneous population of mesenchymal cells of fetal origin that, with higher proliferation and differentiation plasticity, are able to differentiate into all three germ layers, do not form tumors when injected in vivo, and are free of ethical issues concerning their employment^66^. The in vitro experiments performed on AFSCs revealed that the tested GaFeO#1 and GaFeO#2 have good compatibility with eukaryotic cells. The MTT assay demonstrated that human AFSCs present normal metabolism and growth in the presence of GaFeO#1 and GaFeO#2, with the measured values of absorbance at 570 nm being similar to those of control cells after 72 h of incubation (Fig. 13). A slight increase in proliferation is observed at 8.3 mg/mL and 16.66 mg/mL of GaFeO#2 NPs.

**Figure 14:**
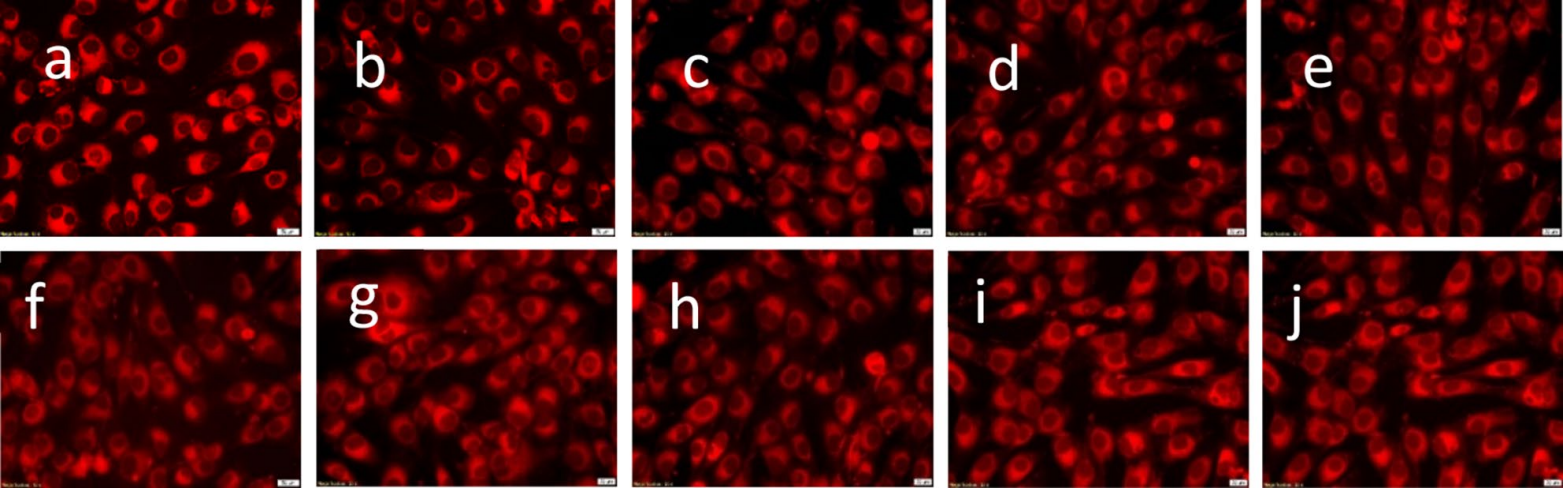
Biocompatibility assessment of GaFeO#1 and GaFeO#2 NPs through fluorescence microscopic images of AFSC monolayers after 5 days: (**a**,**f**) control cells; (**b**) GaFeO#1—3.3 mg/mL; (**c**) GaFeO#1—8.3 mg/mL; (**d**) GaFeO#1—16.66 mg/mL (**e**) GaFeO#1—33.33 mg/mL; (**g**) GaFeO#2—3.3 mg/mL; (**h**) GaFeO#2—8.3 mg/mL; (**i**) GaFeO#2—16.66 mg/mL; (**j**) GaFeO#2—33.33 mg/mL. The all scale bars are 20 μm.

For a better analysis of biocompatibility, we used a second method to assess cytotoxicity. Thereby, fluorescence microscopy using RED CMTPX fluorophore as a cell tracker showed that AFSCs are viable and exhibit normal growth and proliferation capacity in the presence of GaFeO#1 and GaFeO#2 NPs (Fig. 14a–j). The results obtained after 5 days of incubation indicate normal morphology of human AFSCs. The shape and adherence properties are comparable to those of the control cells (Fig. 14a–f). No dead cells or cell fragments are evidenced in the presence of Ga_0.9_Fe_2.1_O_4_ NPs, suggesting that the cells are viable. These observations sustain the fact that the GaFeO#1 and GaFeO#2 NPs are safe for use in further tissue engineering applications.

**Figure 15:**
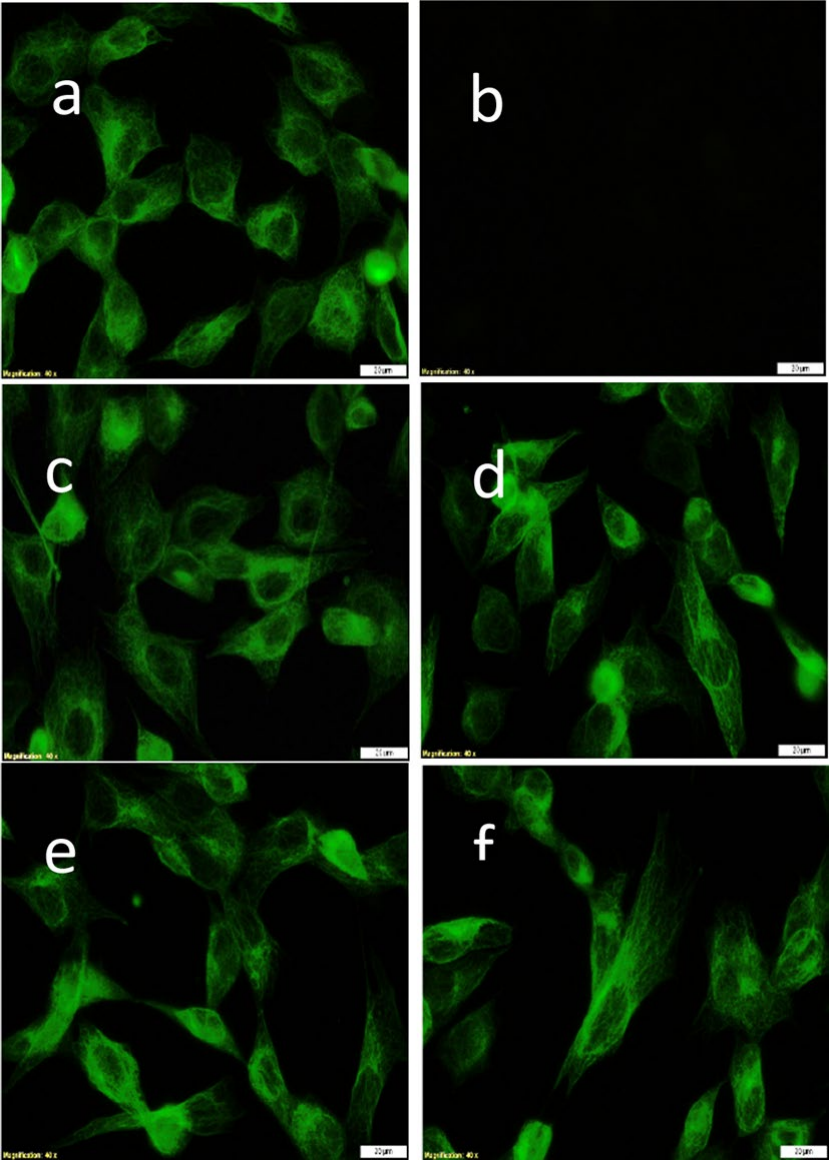
Representative fluorescence images of tubulin filaments of AFSC after 72 h of incubation in the presence of (**c**) GaFeO #1—8.3 mg/mL; (**d**) GaFeO #2—8.3 mg/mL; (**e**) GaFeO #1—33.33 mg/mL; (**f**) GaFeO #2—33.33 mg/mL. Here (**a**) represents the control cells and (**b**) is the negative control-lacking primary antibody. The scale bars are 20 μm.

Furthermore, the biocompatibility of GaFeO#1 and GaFeO#2 NPs was investigated in a more complex manner, by evaluated the tubulin cytoskeleton organization using immunocytochemistry. The results are shown in Fig. 15. The control cells display a normal fibroblastic morphology with a fusiform shape, which enables a good cell attachment, spreading and motility as seen in Fig. 15a. Figure 15c–f represent the human AFSC in the presence of GaFeO#1 and GaFeO#2 NPs. They show no major changes in tubulin filaments reorganization. Nevertheless a slight elongation could be observed at 8.3 mg/mL and 33.33 mg/mL concentrations for both compounds. They preserve their initial architecture, with elongated extensions suggesting an active cytoskeleton represented by the network of microtubules, actin and intermediated filaments of proteins.

To conclude, all the assays showed that GaFeO#1 and GaFeO#2 NPs are biocompatible with human AFSC for the tested concentrations.

## Conclusions

In summary we have prepared ultra-small NPs of Ga_0.9_Fe_2.1_O_4_, below 6 nm, by two different methods: thermal decomposition and microwave assisted process. In both cases acetylacetonates were used as metal precursors and triethylene glycol as high boiling solvent and surfactant. The resulted products are different from the common ferrite compound through the cation distribution and stoichiometry with Ga^3+^ in octahedral sites and only Fe^2+^ in the tetrahedral ones. This is sustained by XRD, XPS, ICP-AES and magnetization measurements. The presence of a TEG layer on the nanoparticle surface is responsible for the long-term NPs’ stability in different solvents, such as: ethanol, hexane and water. The TD method assures the formation of crystalline, free of agglomeration NPs with a ferromagnetic coupling inside the matrix, while the particles obtained using MW method are smaller in size with a low degree of crystallinity and contain a large fraction of non-magnetic NPs as well as gallium suboxide. Thus, even if it is more laborious, the TD method produces GaFeO NPs with better quality with a good magnetic ordering. Also, the biocompatibility assessment showed that NPs are biocompatible with human AFSC. In vitro experiments on amniotic mesenchymal stem cells showed that the cellular metabolism of the cells is active in the presence of Ga_0.9_Fe_2.1_O_4_ NPs being biocompatible with human AFSC. Additionally, the immunohistochemistry demonstrated that AFSC preserve a normal morphology, with a normal fibroblast phenotype that suggest the presence of an active cytoskeleton.

These results bring new insights of the properties of superparamagnetic ultra-small NPs of Ga_0.9_Fe_2.1_O_4_ and may lead to the development of new biomedical applications. Based on the presented results, the synthesized Ga_0.9_Fe_2.1_O_4_ NPs show targeted characteristics and properties for their potential applications in biochemical areas.

### Supplementary Information

Supplementary Information.

## Data Availability

All data generated or analysed during this study are included in this article.
