# Supplementary material for: Insight into synthesis and characterisation of Ga0.9Fe2.1O4 superparamagnetic NPs for biomedical applications
Source: Sci Rep. 2023 Oct 24;13:18175. doi: 10.1038/s41598-023-45285-y (PMC10598038; doi:10.1038/s41598-023-45285-y)
Supplement: Supplementary file 1 — Supplementary Information. [file 41598_2023_45285_MOESM1_ESM.docx]

**Supplementary information**

**for**

**Insight into synthesis and characterization of Ga_0.9_Fe_2.1_O_4_ superparamagnetic NPs for biomedical applications**

Amalia Mesaros, Alba Garzón-Manjón, Mircea Nasui, Rares Bortnic, Bogdan Stefan Vasile, Otilia Ruxandra Vasile, Florin Iordache, Cristian Leostean, Lelia Ciontea, Josep Ros, Ovidiu Pana^*^

* Corresponding author

1. TEM images representing different stages of the NPs nucleation process. They are correlated with TEM images – Fig 1 and XRD patterns recorded at different dwell times - Fig. 3(a) from the main text.


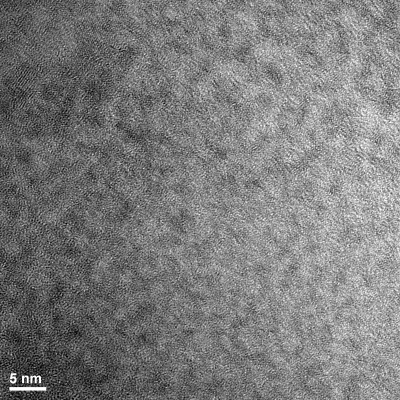


(a)


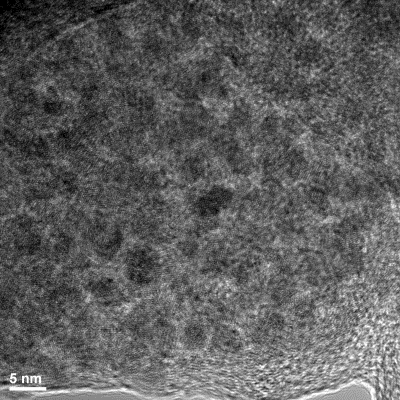


(b)

Figure S1. TEM and HRTEM images for the NPs synthesized by solvothermal approach at different reaction times namely 0 (a) and 1.5 h (b).

2.1. A Rietveld refinement was made for the GaFeO#1 sample. The file used for Ga_0.9_Fe_2.1_O_4_ spinel structure was PDF 074-2226. For the GaFeO#2 sample, where a lot of amorphous phase is present, the fitting convergence could not be reached. The fitting of XRD patterns, as resulted from Rietveld analysis, is shown in Figure S2.

The convergence of the fitting process is represented by the different figures of merit whose values are given in Table S1.

Table S1. Values of figure of merit as resulted from Rietveld analysis of XRD patterns for GaFeO#1 sample.

| Data set name | Rwp | Rp | Re | S | Chi^2 | Maximum shift/e.s.d. |
| --- | --- | --- | --- | --- | --- | --- |
| Ga Fe O | 4.04 | 3.13 | 3.13 | 1.2911 | 1.6669 | 0.09 |





Figure S2 The fit of XRD patterns as resulted from Rietveld analysis.

2.2. The lattice parameters as resulted from the analysis of XRD patterns for sample GaFeO#1 are given in Table S2. The demined value of the lattice constant a=b=c is by ~0.07% smaller than the nominal value of 8.377 Å provided by the mentioned PDF.

Table S2 Lattice parameters as resulted from the diffraction patterns.

| Phase name | a(A) | b(A) | c(A) | α(deg) | β (deg) | γ (deg) | V(Å^3^) |
| --- | --- | --- | --- | --- | --- | --- | --- |
| Iron Gallium Oxide | 8.3713(3) | 8.3713(3) | 8.3713(3) | 90.00000 | 90.00000 | 90.00000 | 586.64(3) |

3. XPS line positions and normalized integral intensities

The XPS spectra of the C 1*s* core-level assigned to carbon residues onto the surface of the NPs are shown in Figures S3 and S4 respectively.





Figure S3. XPS spectrum of C 1s core-level for GaFeO#1 sample.





Figure S4. XPS spectrum of C 1s core-level for GaFeO#2 sample. One can observe that the C=O position is missing here.

Tables S3 and S4 summarize the XPS core-level line positions, half-widths, raw areas and normalized areas for GafEo#1 and GaFeO#2 samples respectively. The values of the normalized areas are proportional to the number of atoms of the corresponding species. Here RSF represents the Relative Sensitivity Factor, T the Transmission coefficient and MFP is the Mean Free Path of the ejected electrons. Their values were taken from CASA software data base. In composite materials, for quantitative analysis, the normalized areas should be divided the escape lengths which are specific for a given material. One can observe that for the microwave prepared sample the C=O line in the C 1*s* spectrum is absent. This type of bonding is disrupted in the MW preparation route.

Table S3 XPS core-level characteristics for sample GaFeO#1.

| Core-level name | BE  (eV) | FWHM (eV) | Raw Area  (eV s^-1^) | Normalized Area /(RSF*T*MFP) |
| --- | --- | --- | --- | --- |
| Ga 3d (5/2) – (1+) | 17.07 | 1.36 | 1434 | 447 |
| Ga 3d (3/2) – (1+) | 17.47 | 1.43 | 957 | 433 |
| Ga 3d (5/2) – (3+) | 18.79 | 2.38 | 15689 | 4892 |
| Ga 3d (3/2) – (3+) | 19.19 | 2.50 | 10465 | 4747 |
| Fe 2p (3/2) – (2+) | 709.15 | 4.75 | 252089 | 5443 |
| Fe 2p (1/2) – (2+) | 722.41 | 4.99 | 126045 | 5270 |
| Fe 2p (3/2) – (3+) | 711.75 | 6.89 | 277298 | 5992 |
| Fe 2p (1/2) – (3+) | 725.01 | 7.23 | 138649 | 5801 |
| Fe 2p sat 1 | 715.01 | 6.00 | - | - |
| Fe 2p sat 2 | 718.38 | 6.88 | - | - |
| Fe 2p sat 3 | 729.03 | 6.00 | - | - |
| Fe 2p sat 4 | 733.14 | 4.81 | - | - |
| C 1s; C‒C, CH | 284.6 | 4.5 | 14130 | 2971 |
| C 1s, C‒O | 288.1 | 1.8 | 990 | 208 |
| C 1s, C=O | 289.9 | 2.9 | 830 | 175 |

Table S4 XPS core-level characteristics for sample GaFeO#2.

| Core-level name | BE  (eV) | FWHM (eV) | Raw Area  (eV s^-1^) | Normalized Area /(RSF*T*MFP) |
| --- | --- | --- | --- | --- |
| Ga 3d (5/2) – (1+) | 17.82 | 1.5 | 5088 | 1587 |
| Ga 3d (3/2) – (1+) | 18.27 | 1.6 | 3395 | 1540 |
| Ga 3d (5/2) – (3+) | 19.4 | 1.8 | 9304 | 2903 |
| Ga 3d (3/2) – (3+) | 19.86 | 1.6 | 6205 | 2817 |
| Fe 2p 2+ 3/2 | 708.76 | 4.6 | 148188 | 3203 |
| Fe 2p 2+ 1/2 | 722.02 | 4.9 | 74093.8 | 3101 |
| Fe 2p 3+ 3/2 | 710.2 | 7.3 | 163006 | 3525 |
| Fe 2p 3+ 1/2 | 723.46 | 7.7 | 81503 | 3413 |
| Fe 2p sat 1 | 714.5 | 5.5 | - | - |
| Fe 2p sat 2 | 717.58 | 5.0 | - | - |
| Fe 2p sat 3 | 727.99 | 3.2 | - | - |
| Fe 2p sat 4 | 730.64 | 4.6 | - | - |
| C 1s, C‒C, CH | 284.6 | 3.9 | 23158 | 4873 |
| C 1s, C‒O | 288.1 | 2.1 | 2108 | 444 |

4. Magnetic characterization

In the case of the GaFeO#2 sample, due to the very small size of some NPs, there is an undetermined content of the ferromagnetic phase that behaves superparamagnetic. An example of fitting, in the case of this sample, is shown in Figure S5. The resulting fit parameters, M_S_, D_0_ and σ, are 8.0 emu/g, 2.5 nm and 0.65, respectively for a GaFeO#2 mass concentration of 0.5%. The D_0_ and σ values are too high and have no physical meaning for this sample. Depending on the concentration of superparamagnetic material (diameters greater than 2.5-3 nm) to be considered, different contradictory solutions are obtained. Practically the concentration of the very small NPs cannot be determined. Thus, due to the small size of some NPs ensemble, the applicability limit of this model is reached.

| 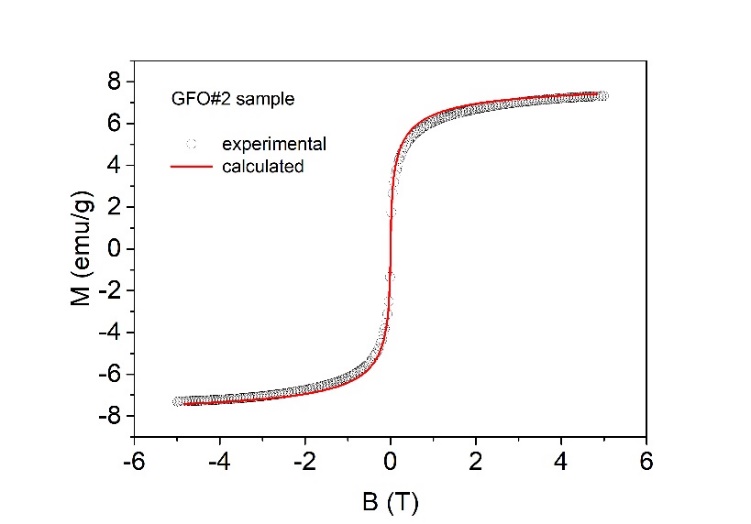 |
| --- |
| Figure S5. Example of fitting of the magnetization as a function on the applied magnetic field in case of sample GaFeO#2. |
